# A non-standard numerical scheme for an alcohol-abuse model with induced-complications

**DOI:** 10.1016/j.heliyon.2023.e22263

**Published:** 2023-11-13

**Authors:** Eric Abaa Baba Sandow, Baba Seidu, Stephen Abagna

**Affiliations:** a Department of Mathematics, Nalerigu Senior High School, Nalerigu NER, Ghana; b Department of Mathematics, C. K. Tedam University of Technology and Applied Sciences, Navrongo, Ghana; c Department of Mathematics, Sirigu Senior High School, Sirigu UE/R, Ghana

**Keywords:** 92D30, 92D25, 37N25, 34D20, 92B05, Substance-abuse, Non-standard finite difference, Bifurcation analysis, Stability analysis

## Abstract

The prevalence of alcohol-related fatalities worldwide is on the ascendancy not only Ghana, but worldwide. Although the ramifications of alcohol consumption have been the subject of several studies, alcoholism remains a serious concern in public health. This study investigates the dynamics of alcoholism in a population with consumption-induced complications using a deterministic Modelling framework. Using a novel technique, we determined a threshold parameter $R_{0}$ which we call the basic alcohol-abuse initiation number which is similar to the basic reproduction number for infectious diseases. The model has two mutually-exclusive fixed points whose existence depend on whether or not the $R_{0}$ is less or greater than unity. Global asymptotic stability of the alcohol-abuse-free fixed point is shown to be associated with $R_{0}\leq 1$. Further, forward bifurcation is observed to occur at $R_{0}=1$, indicating the possibility of eradication of the phenomenon of alcoholism if $R_{0}$ can be kept below unity over a sufficiently long period of time. Sensitivity analysis also revealed that the probability of initiation into alcohol-abuse by moderate drinkers ($\beta_{1}$), followed by the probability of initiation into alcohol-abuse by heavy drinkers ($\beta_{2}$) are the most the parameters with the most influence on $R_{0}$ and consequently on alcohol-abuse persistence. A non-standard finite difference scheme is also developed to numerically simulate the model so as to demonstrate the findings derived from the analysis and also to observe the impact of some epidemiological factors on the dynamics of alcohol-abuse.

## 1 Introduction

Alcohol drinking is one of the most dangerous activities that young people erroneously regard as fun or enjoyment. Besides, it was falsely recommended in 2019 that, alcohol was very effective in the treatment of corona virus (COVID-19) and this led to a lot of alcohol poisoning [1]. The initiation into alcohol-abuse should be considered as a public concern because there is no such thing as a safe consumption level [2]. The rate of alcohol consumption during the outbreak of COVID-19 was a little higher for males as compared to the previous 2–3 years and significantly higher in females [3]. It is estimated that alcohol consumption is responsible for the death of about 2.5 million people globally each year [4]. The World Health Organization (WHO) indicates that, alcohol-abuse is expected to cause approximately 60 types of disorders including liver cancer, liver cirrhosis, heart disease, esophageal cancer, homicide, pancreatitis, stroke, epileptic, neuropathy disorders, brain disorder, fatty liver problems, hepatic, type 2 diabetes, hypertension and dyspepsia.

Mathematics has played an important role in researching into many disciplines. Among these are its application to disease control. Researchers have developed many mathematical models to predict and explain the effects of epidemics. However, the world still battles with a lot of diseases which are caused by either natural or artificial factors. All these explain why mathematicians have to do more research in building mathematical models to help reduce the risk of being exposed to epidemic diseases. Mathematical models have demonstrated high potential in helping to improve our knowledge of the dynamics of infectious agents and diseases. With alcoholism being a public health concern, many researchers have concentrated on building models to study the dynamics of alcoholism. Sa'nchez et al. [5] proposed an alcohol drinking model with recovery relapse and described the transmission dynamics of alcohol within the context of the classic SIR model. Huo and Song [6] presented an alcohol-abuse model to study binge drinking among youth. Another deterministic alcohol-abuse model was in [7] in order to gain more insight into this health concern and social phenomenon and to determine the most cost-effective way of addressing the canker. Xiang et al. [8] studied the global stability of an alcohol-use model with public health awareness campaign. Khajji et al. [9] developed a model with liver complications that specifically deals with educating alcohol-abusers and giving treatment to those with disorders. Pe'rez [10] presented a non-linear differential equation model, describing the difficulties and problems associated with alcohol consumption habits among college students in Colombia. Khajji et al. [11] proposed a model in order to look at the interaction between different groups of people with different levels of alcohol consumption. They focused on treatment of heavy alcoholics drinkers. In [12] a discrete mathematical model was presented to elucidate the interaction between the various compartments of drinkers. They also sought to determine optimal strategies for the minimization of the population of drinkers and maximization of acceptance by heavy drinkers to seek treatment. On the basis of the above research works and several others, the purpose of this study is to put forth a mathematical model, suitable for describing the dynamics of alcohol abuse to include the quitting and recovery rate moderate alcohol users, which is often neglected by most research on alcohol-use models. In this study, the effect of moderate drinkers, heavy drinkers, and individuals with complications recovering is discussed and also, the likelihood of the recovered individuals rejoining the chain of drinking is considered. Most researches involving infectious diseases and substance-abuse often use explicit methods to numerically solve the models. However, it is known that explicit methods such as the Euler and Runge-Kutta methods have inherent challenges including poor stability and efficiencies, which may give wrong dynamical behavior which are not inherent in the studied dynamical systems [13]. For instance, use of the explicit methods may indicate bifurcations and chaos which the system may not really posses. The implicit methods have the ability to avoid these contrived dynamics while maintaining their efficiency and stability. This makes the implicit methods a better choice over their explicit counterpart. The non-standard finite difference methods developed in [14], [15] have motivated several other schemes and the use of same in solving numerical schemes. Following these works, a non-standard finite difference scheme is developed to use in the numerical experimentation on the model.

We structure the remainder of the paper in the following manner: Section 2 presents the development of the Mathematical model in focus. Qualitative analysis including stability of equilibrium points, bifurcation analysis, and sensitivity analysis of the model are presented in Section 3. In section 4, we develop a non-standard numerical system to be used for simulation of the model. In Section 5, the model is solved using the developed numerical scheme and the discussion of findings of the study are presented. Finally, the conclusions of the study are captured in section 6.

## 2 Development of the alcohol-abuse model

**Figure 1:**
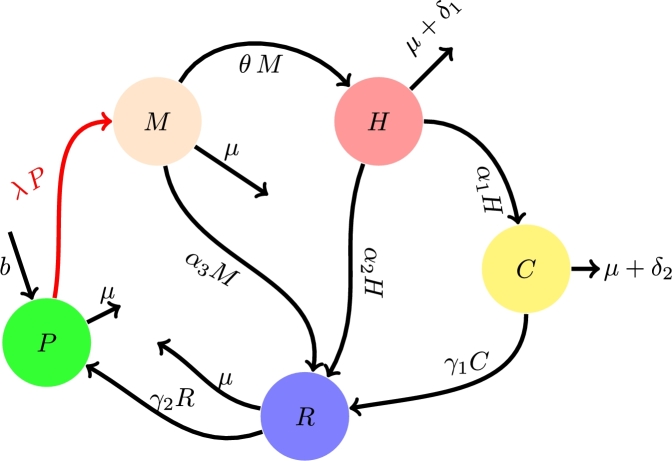
Schematic diagram of the dynamics of alcohol-abuse with induced-complications.

In this section, the deterministic mathematical model of interest is developed to elucidate the dynamics of alcoholism with induced-complications. We categorize the population into five distinct and non-overlapping compartments: The compartment of Potential drinkers (P) denotes people who do not consume alcohol but are prone to adopting alcohol consumption behavior; Moderate alcohol abusers (M) represents individuals who slightly consume alcohol and have the ability to control the intake of alcohol; Heavy alcohol abusers (H) denotes people who are obsessive to alcohol consumption (unable to control the intake of alcohol) and; Alcohol-abusers who have developed alcohol-induced complications (C) representing people who develop disorders such as liver cirrhosis, brain disorders, neuropathy complications, fibrosis, pancreatitis, and alcoholic hepatitis. We assume that all inflows into the population are Potential drinkers at the rate of *b*. We define $\lambda =\frac{\beta_{1}M(t)+\beta_{2}H(t)}{N}$ (similar to force of infection) as the rate at which potential drinkers adopt alcohol-consumption behavior due to contact with alcohol-consumers. The parameter, $\beta_{1}$ is the probability that a potential drinker starts to abuse alcohol as a result of interaction with a moderate drinker, and $\beta_{2}$ is the probability that potential drinker begins to abuse alcohol due to interaction with a heavy drinker. The moderate drinkers are assumed to progress into heavy drinking at a rate of *θ* and they quit drinking alcohol at the rate of $\alpha_{3}$. Heavy drinkers are assumed to develop complications at the rate of $\alpha_{1}$ and quit alcohol consumption at the rate of $\alpha_{2}$. Individuals with induced-complications are assumed to recover at the rate of $\gamma_{1}$. Persons who recovered from alcohol-abuse are assumed to rejoin the compartment of potential drinkers at the rate of $\gamma_{2}$ per unit time. The parameters *μ*, $\delta_{1}$, and $\delta_{2}$ respectively represent death rate due to nature, death rate do to alcohol-abuse among heavy drinkers, and death rate associated with complications caused by alcohol-abuse. The general dynamics of alcohol abuse with induced-complications are depicted in the schematic diagram in Fig. 1.

The alcohol-abuse model described so far is thus presented by the following coupled ordinary differential equations.

(1) $$\begin{array}{c} \frac{dP}{dt}=b+\gamma_{2}R-\left(\lambda +\mu \right)P,\\ \frac{dM}{dt}=\lambda \;P-\left(\mu +\theta +\alpha_{3}\right)M,\\ \frac{dH}{dt}=\theta M-\left(\alpha_{1}+\alpha_{2}+\mu +\delta_{1}\right)H,\\ \frac{dC}{dt}=\alpha_{1}H-\left(\gamma_{1}+\mu +\delta_{2}\right)C,\\ \frac{dR}{dt}=\alpha_{2}H+\alpha_{3}M+\gamma_{1}C-\left(\mu +\gamma_{2}\right)R. \end{array}\}$$

In subsequent discussions, where necessary, the following conventions will be used.

$$\begin{array}{cccc} \xi_{1}=\mu +\theta +\alpha_{3},\; & \xi_{2}=\alpha_{1}+\alpha_{2}+\mu +\delta_{1},\; & \xi_{3}=\gamma_{1}+\mu +\delta_{2},\; & \xi_{4}=\mu +\gamma_{2}. \end{array}$$

The description of the model parameters is summarized in Table 1. The baseline values of the parameters used later for simulation purposes are also presented in Table 1. Some qualitative properties of the model are presented in the following section.

**Table 1:** Description and baseline values of the model parameters.

Par.	Description	Value	Source
*b*	Recruitment rate into the population	65	[9]
*μ*	Natural death rate	0.065	[9]
*β*_1_	Probability of developing alcohol-consumption behavior per contact with a moderate drinker	0.02	[9]
*β*_2_	Probability of developing alcohol-consumption behavior per contact with a heavy drinker	0.01	Assumed
*θ*	Rate of transition from moderate drinking to heavy drinking	0.14	Assumed
*δ*_1_	Rate of death resulting from high alcohol consumption	0.002	[9]
*α*_1_	Rate of development of alcohol-induced complication	0.02	[9]
*α*_2_	Rate of recovery from heavy alcohol consumption	0.001	[9]
*δ*_2_	Death rate resulting from alcohol-induced complication	0.002	[9]
*γ*_1_	Recovery rate of individuals in the complication compartment	0.001	[9]
*γ*_2_	Rate of relapse of Recovered persons into susceptibility	0.0002	Assumed
*α*_3_	Recovery rate from moderate drinking	0.0001	Assumed

## 3 Qualitative properties

It is easy to observe that the functions in the right-hand-side of (1) are Schlitz continuous, which is a necessary condition for Picard's existence theorem [16]. Therefore, we can conclude that, the alcohol-abuse model (1) has a unique solution.

The epidemiological and mathematical well-posedness of the model are presented in Theorem 3.1.

Theorem 3.1 *With non-negative initial conditions, the solution of the alcohol-abuse model*(1)*are always non-negatives for all time,*t>0*.*

Proof Assume that for some time $t_{1}$ with $t_{1}>t>0$ we have  and P(t)>0,M(t)>0,H(t)>0,C(t)>0,R(t)>0. Then from the first equation of (1), we have , which contradicts our assumption. Therefore P(t)>0. Similar arguments show that the remainder of the state variables are also non-negative, concluding the proof. □

The set of epidemiologically-reasonable solutions of the alcohol-abuse model (1) is named as the feasible region, wherein all solutions of the model with reasonable initial conditions are contained. We define this set in Lemma 3.1.

Lemma 3.1 *Future solution of the alcohol-abuse model*(1)*with non-negative initial conditions are all non-negative and contained in*$\Omega =\{\left(P,\;M,\;H,\;C,\;R\right)\in R_{+}^{5}|N\leq \frac{b}{\mu }\}$*, which is positively invariant.*

Proof Adding all equations in (1) gives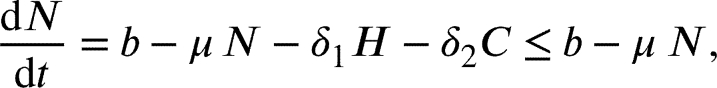 whose solution is given by

$$N\left(t\right)\leq N\left(0\right)\exp\left(-\mu \;t\right)+\frac{b}{\mu }\left(1-\exp\left(-\mu \;t\right)\right).$$

Therefore, $\underset{t\to \infty }{\lim\;\sup}\;N\left(t\right)\leq \frac{b}{\mu }$.

Therefore, the solutions of the alcohol-abuse model (1) are uniformly bounded in Ω, completing the proof. □

### 3.1 Equilibrium points and the basic reproduction number of the alcohol-abuse model (1)

The model (1) has two fixed points; the alcohol-abuse-free equilibrium ($E_{A0}$) and the alcohol-abuse-persistent equilibrium $E^{⁎}$.

The alcohol-abuse-free equilibrium given by $E_{A0}=\left(\frac{b}{\mu },\;0,\;0,\;0,\;0\right)$ is obtained by setting M=H=C=R=0 in (1).

The basic reproduction number denoted as $R_{0}$ is an epidemiological quantity used to describe the mean number of secondary infections that arises from the introduction of a single infectious agent into an otherwise completely Susceptible population. In this study, $R_{0}$ is named *basic alcohol-abuse initiation number*. We define $R_{0}$ as the average number of alcohol-consumers that are initiated by a single alcohol-abuser introduced into an otherwise Potential drinkers population. In this paper, we adopt the Jacobian-Determinant method of [17] to determine $R_{0}$ of the alcohol-abuse model (1). Following [17], the infected sub-system is given as follows:

(2) $$\begin{array}{c} \frac{dM}{dt}=\lambda \;P-\xi_{1}M,\\ \frac{dH}{dt}=\theta M-\xi_{2}H,\\ \frac{dC}{dt}=\alpha_{1}H-\xi_{3}C. \end{array}\}$$

The Jacobian of the infected sub-system (2) evaluated at $E_{A0}$ is given by

$$J_{I}\left(E_{A0}\right)=\left[\begin{array}{ccc} \beta_{1}-\xi_{1}\; & \beta_{2}\; & 0\\ \theta \; & -\xi_{2}\; & 0\\ 0\; & \alpha_{1}\; & -\xi_{3} \end{array}\right],$$

whose determinant is given by

$$Det\left(J_{I}\left(E_{A0}\right)\right)=\left(\theta \beta_{2}+\xi_{2}\beta_{1}-\xi_{1}\xi_{2}\right)\xi_{3}.$$

Following the formulation in [17], the basic alcohol-abuse initiation number is given by

$$R_{0}=\frac{\beta_{1}}{\xi_{1}}+\frac{\beta_{2}\;\theta }{\xi_{1}\xi_{2}}.$$

The alcohol-abuse persistent equilibrium point can be shown to be given by $E^{⁎}=\left(P^{⁎},\;M^{⁎},\;H^{⁎},\;C^{⁎},\;R^{⁎}\right)$, where

(3) $$\begin{array}{c} P^{⁎}=\frac{b}{\mu }\left(\frac{\hbar_{0}-\theta \xi_{4}\left(\alpha_{1}\delta_{2}+\delta_{1}\xi_{3}\right)}{R_{0}\hbar_{0}-\theta \xi_{4}\left(\alpha_{1}\delta_{2}+\delta_{1}\xi_{3}\right)}\right),\\ M^{⁎}=\frac{b\xi_{2}\xi_{3}\xi_{4}\left(R_{0}-1\right)}{R_{0}\hbar_{0}-\theta \xi_{4}\left(\alpha_{1}\delta_{2}+\delta_{1}\xi_{3}\right)},\\ H^{⁎}=\frac{b\theta \xi_{3}\xi_{4}\left(R_{0}-1\right)}{R_{0}\hbar_{0}-\theta \xi_{4}\left(\alpha_{1}\delta_{2}+\delta_{1}\xi_{3}\right)},\\ C^{⁎}=\frac{b\theta \xi_{4}\alpha_{1}\left(R_{0}-1\right)}{R_{0}\hbar_{0}-\theta \xi_{4}\left(\alpha_{1}\delta_{2}+\delta_{1}\xi_{3}\right)},\\ R^{⁎}=\frac{b\left(\theta \alpha_{1}\gamma_{1}+\theta \alpha_{2}\xi_{3}+\alpha_{3}\xi_{2}\xi_{3}\right)\left(R_{0}-1\right)}{\left(R_{0}\hbar_{0}-\theta \xi_{4}\left(\alpha_{1}\delta_{2}+\delta_{1}\xi_{3}\right)\right)},\\ \hbar_{0}=\left(\theta \alpha_{1}\gamma_{1}+\theta \alpha_{2}\xi_{3}+\alpha_{3}\xi_{2}\xi_{3}\right)\gamma_{2}+\xi_{1}\xi_{2}\xi_{3}\xi_{4}. \end{array}\}$$

### 3.2 Local stability of fixed points of the alcohol-abuse model (1)

Theorem 3.2, Theorem 3.3 present the results on local stability of the fixed points of the model (1). The Lyapunov second technique is employed to study the local stability of the equilibria.

The Jacobian matrix of the alcohol-abuse model (1) is given by

$$J=\left[\begin{array}{ccccc} \frac{\lambda P}{N}-\lambda -\mu \; & -\left(\frac{\beta_{1}}{N}-\frac{\lambda }{N}\right)P\; & -\left(\frac{\beta_{2}}{N}-\frac{\lambda }{N}\right)P\; & \frac{\lambda P}{N}\; & -\gamma_{2}+\frac{\lambda P}{N}\\ \lambda -\frac{\lambda P}{N}\; & -\xi_{1}+\frac{\beta_{1}P}{N}-\frac{\lambda P}{N}\; & \frac{\beta_{2}P}{N}-\frac{\lambda P}{N}\; & -\frac{\lambda P}{N}\; & -\frac{\lambda P}{N}\\ 0\; & \theta \; & -\xi_{2}\; & 0\; & 0\\ 0\; & 0\; & \alpha_{1}\; & -\xi_{3}\; & 0\\ 0\; & \alpha_{3}\; & \alpha_{2}\; & \gamma_{1}\; & -\xi_{4} \end{array}\right].$$

The characteristic polynomial corresponding to the alcohol-abuse-free critical point $E_{A0}$ is given by

(4) $$\begin{array}{c} \chi^{2}+\left(\xi_{1}\left(1-R_{0}\right)+\xi_{2}+\frac{\beta_{2}\theta }{\xi_{2}}\right)\chi +\xi_{1}\xi_{2}\left(1-R_{0}\right)=0 \end{array}$$

Now, the coefficients of equation (4) are positive whenever $R_{0}<1$, so that by the Routh Hurwitz criterion, all the zeros of the polynomial in equation (4) will have negative real parts. This establishes the following result.

Theorem 3.2 *The alcohol-abuse-free fixed point,*$E_{A0}$*is locally asymptotically stable whenever*$R_{0}<1$*. Otherwise,*$E_{A0}$*is unstable.*

The characteristic polynomial of J evaluated at $E^{⁎}$ is given by;

(5) $$\chi^{5}+\Phi_{4}\chi^{4}+\Phi_{3}\chi^{3}+\Phi_{2}\chi^{2}+\Phi_{1}\chi +\Phi_{0}=0,$$

where,

$$\begin{array}{c} \Phi_{4}=\lambda^{⁎}+\mu +\xi_{1}+\xi_{2}+\xi_{3}+\xi_{4}-\frac{\beta_{1}}{R_{0}},\\ \Phi_{3}=\left(\xi_{1}+\xi_{2}+\xi_{3}+\xi_{4}\right)\left(\lambda^{⁎}+\mu \right)+\xi_{1}\left(\xi_{2}+\xi_{3}+\xi_{4}\right)+\xi_{2}\xi_{3}+\xi_{2}\xi_{4}+\xi_{3}\xi_{4}-\frac{1}{R_{0}}\left[\left(\mu +\xi_{2}+\xi_{3}+\xi_{4}\right)\beta_{1}+\theta \beta_{2}\right],\\ \Phi_{2}=[\left(\mu \theta +\mu \alpha_{3}+\mu \xi_{2}+\mu \xi_{3}+\mu \xi_{4}+\theta \alpha_{1}+\theta \alpha_{2}+\theta \xi_{3}+\theta \xi_{4}+\alpha_{3}\xi_{2}+\alpha_{3}\xi_{3}\right)\frac{1}{R_{0}}\\ \;\;+\left(\alpha_{3}\gamma_{2}+\xi_{1}\xi_{2}+\xi_{1}\xi_{3}+\xi_{1}\xi_{4}\right)\left(1-\frac{1}{R_{0}}\right)+\xi_{2}\xi_{3}+\xi_{2}\xi_{4}+\xi_{3}\xi_{4}]\lambda^{⁎}-\frac{\beta_{1}}{R_{0}}\left(\mu \xi_{3}+\mu \xi_{4}+\xi_{3}\xi_{4}\right)\\ \;\;+\left(\mu \xi_{3}+\mu \xi_{4}+\xi_{3}\xi_{4}\right)\left(\xi_{2}+\xi_{1}\right)+\mu \xi_{3}\xi_{4};\\ \Phi_{1}=\{[\left(\left(\alpha_{1}+\alpha_{2}+\xi_{3}+\xi_{4}\right)\mu +\gamma_{1}\alpha_{1}+\xi_{4}\alpha_{1}+\alpha_{2}\xi_{3}+\xi_{3}\xi_{4}\right)\theta +\left(\left(\xi_{2}+\xi_{3}\right)\mu +\xi_{2}\xi_{3}\right)\alpha_{3}+\left(\xi_{2}\xi_{3}+\xi_{2}\xi_{4}+\xi_{3}\xi_{4}\right)\mu ]\frac{1}{R_{0}}\\ \;\;+\left(\alpha_{2}\gamma_{2}\theta +\alpha_{3}\gamma_{2}\xi_{2}+\alpha_{3}\gamma_{2}\xi_{3}+\xi_{1}\xi_{2}\xi_{3}+\xi_{1}\xi_{2}\xi_{4}\right)\left(1-\frac{1}{R_{0}}\right)\}\lambda^{⁎}\\ \;\;+\left(\xi_{1}\xi_{3}\xi_{4}+\xi_{2}\xi_{3}\xi_{4}\right)\left(\lambda^{⁎}+\mu \right)-\frac{1}{R_{0}}\xi_{3}\xi_{4}\left(\lambda^{⁎}\xi_{1}+\mu \beta_{1}\right);\\ \Phi_{0}=[\frac{1}{R_{0}}\left(\left(\left(\alpha_{2}+\xi_{4}\right)\theta +\xi_{2}\left(\xi_{4}+\alpha_{3}\right)\right)\xi_{3}+\theta \alpha_{1}\left(\gamma_{1}+\xi_{4}\right)\right)\mu +\left(\left(\left(\theta \alpha_{2}+\alpha_{3}\xi_{2}\right)\xi_{3}+\theta \alpha_{1}\gamma_{1}\right)\gamma_{2}+\xi_{1}\xi_{2}\xi_{3}\xi_{4}\right)\left(1-\frac{1}{R_{0}}\right)]\lambda^{⁎}. \end{array}$$

The following results follows from application of the Routh-Hurwitz criteria on equation (5).

Theorem 3.3 *The following conditions guarantee the local asymptotic stability of the alcohol-abuse-persistent fixed point*$E^{⁎}$*of model*(1)*:*

$$\begin{array}{c} \Phi_{4}>0,\;\Phi_{0}>0,\;\frac{\Phi_{3}\Phi_{4}-\Phi_{2}}{\Phi_{4}}>0,\;\frac{\Phi_{1}\Phi_{4}^{2}-\Phi_{4}\left(\Phi_{2}\Phi_{3}+\Phi_{0}\right)+\Phi_{2}^{2}}{\Phi_{2}-\Phi_{3}\Phi_{4}}>0,\;\frac{\left(\left(\Phi_{2}\Phi_{3}+2\Phi_{0}\right)\Phi_{1}-\Phi_{0}\Phi_{3}^{2}\right)\Phi_{4}+\Phi_{0}\Phi_{2}\Phi_{3}-\left(\Phi_{1}^{2}\Phi_{4}^{2}+\Phi_{1}\Phi_{2}^{2}+\Phi_{0}^{2}\right)}{\Phi_{4}\left(\Phi_{2}\Phi_{3}+\Phi_{0}\right)-\left(\Phi_{1}\Phi_{4}^{2}+\Phi_{2}^{2}\right)}>0. \end{array}$$

### 3.3 Global stability of $E_{A0}$

In this brief, the global stability of the alcohol-abuse-free fixed point $E_{A0}$ using the first technique of Lyapunov. To determine the Lyapunov function for the model, we adopted the technique described in [17] as follows:

Since the infected sub-system consists of *M*, *H* and *C*, we use a Lyapunov function candidate give by

$$L=KM+K_{2}H+K_{3}C.$$

Recall that,

$$J_{I}\left(E_{A0}\right)=\left[\begin{array}{ccc} \beta_{1}-\xi_{1}\; & \beta_{2}\; & 0\\ \theta \; & -\xi_{2}\; & 0\\ 0\; & \alpha_{1}\; & -\xi_{3} \end{array}\right].$$

Following [17], the infectivity vector for this model is given by $T=\left(\beta_{1},\;\beta_{2},\;0\right)$, so that the Lyapunov coefficients are obtained as follows.

$K_{1}$ is obtained by replacing the first row of $J_{I}\left(E_{A0}\right)$ with T to get.

$$K_{1}=J_{I}^{1}=\left\|\begin{array}{ccc} \beta_{1}\; & \beta_{2}\; & 0\\ \theta \; & -\xi_{2}\; & 0\\ 0\; & \alpha_{1}\; & -\xi_{3} \end{array}\right\|=\xi_{3}\left(\theta \beta_{2}+\xi_{2}\beta_{1}\right)$$

$K_{2}$ is obtained by replacing the second row of $J_{I}\left(E_{A0}\right)$ with T to get.

$$K_{2}=J_{I}^{2}=\left\|\begin{array}{ccc} \beta_{1}-\xi_{1}\; & \beta_{2}\; & 0\\ \beta_{1}\; & \beta_{2}\; & 0\\ 0\; & \alpha_{1}\; & -\xi_{3} \end{array}\right\|=\beta_{2}\xi_{3}\xi_{1}$$

$K_{3}$ is obtained by replacing the third row of $J_{I}\left(E_{A0}\right)$ with T to get.

$$K_{3}=J_{I}^{3}=\left\|\begin{array}{ccc} \beta_{1}-\xi_{1}\; & \beta_{2}\; & 0\\ \theta \; & -\xi_{2}\; & 0\\ \beta_{1}\; & \beta_{2}\; & 0 \end{array}\right\|=0.$$

Therefore, we define the Lyapunov function as $L\left(M,\;H\right)=\xi_{3}\left(\theta \beta_{2}+\beta_{1}\xi_{2}\right)M+\beta_{2}\xi_{1}\xi_{3}H$.

Then 
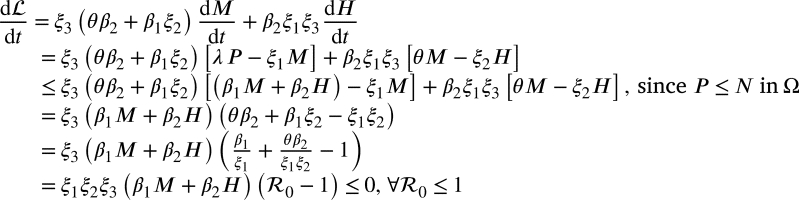
 Thus,  if $R_{0}\leq 1$, with  if and only if M=H=0. Furthermore, the alcohol-abuse-free equilibrium point of the model (1), $E_{A0}$ is the largest invariant compact set in .

From the Lasalle's invariance principle [18], every solution of (1) with initial conditions in Ω converges to $E_{A0}$ as t→∞ whenever $R_{0}\leq 1$. This establishes the following result.

Theorem 3.4 *The alcohol-abuse-free fixed point*$E_{A0}$*of model*(1)*is GAS whenever*$R_{0}\leq 1$*.*

### 3.4 Bifurcation analysis

Let $\beta_{1}^{⁎}=\xi_{1}-\frac{\beta_{2}\;\theta }{\xi_{2}}$ (from $R_{0}=1$) be the bifurcation parameter. Then J evaluated $E_{A0}$ has a simple eigenvalue associated the following right and left eigenvectors: $w={\left(w_{1},\;w_{2},\;w_{3},\;w_{4},\;w_{5}\right)}^{T}\text{ and }v=\left(v_{1},\;v_{2},\;v_{3},\;v_{4},\;v_{5}\right)$ where

$$\begin{array}{cccc} w_{1}=-\frac{1}{\mu }\left(\xi_{1}w_{2}+\gamma_{2}w_{5}\right),\; & w_{3}=\frac{\theta \;w_{2}}{\xi_{2}}\; & w_{4}=\frac{\alpha_{1}\;\theta \;w_{2}}{\xi_{3}\;\xi_{2}},\; & w_{5}=\frac{1}{\xi_{4}}\left(\alpha_{3}+\frac{\alpha_{2}\;\theta }{\xi_{2}}+\frac{\alpha_{1}\;\gamma_{1}\;\theta }{\xi_{3}\;\xi_{2}}\right)\;w_{2},\\ v_{1}=0,\; & v_{3}=\frac{\beta_{2}\;v_{2}}{\xi_{2}},\; & v_{4}=0,\; & v_{5}=0. \end{array}$$

Note that $w_{2}$ and $v_{2}$ can be found using w•v=1 (or equivalently $w_{2}v_{2}=\frac{\xi_{2}^{2}}{\xi_{2}^{2}+\beta_{2}\theta }$).

The bifurcation coefficients are thus obtained (using $a=\sum_{i,j,k=1}^{5}v_{k}w_{i}w_{j}\frac{\partial^{2}f_{k}}{\partial x_{i}\partial x_{j}},\;b=\sum_{i,k=1}^{5}v_{k}w_{i}\frac{\partial^{2}f_{k}}{\partial x_{i}\partial \beta_{1}^{⁎}}$) as

$$a=-\frac{2\;\mu \xi_{2}\hbar_{0}}{b\xi_{3}\xi_{4}\left(\xi_{2}^{2}+\beta_{2}\theta \right)}w_{2},\;\text{ and }b=\frac{\xi_{2}^{2}}{\xi_{2}^{2}+\beta_{2}\theta }.$$

**Figure 2:**
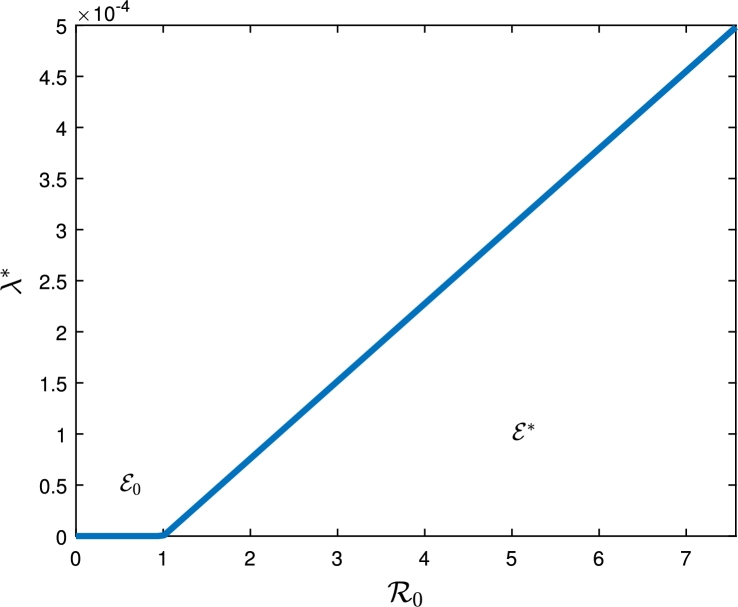
Bifurcation diagram of the alcohol-abuse model (1).

Clearly, a<0 and b>0 and hence, from Theorem 4.1 of [19], the model exhibits forward bifurcation at $R_{0}=1$ as illustrated in Fig. 2.

### 3.5 Sensitivity analysis

We conducted sensitivity analysis to determine the impact of small changes in model parameter values on ($R_{0}$) and the state variables at the endemic equilibrium, $E^{⁎}$. The sensitivity index of variable *X* with respect to a model parameter *p* is computed using the Normalized Forward Sensitivity Index defined as:

$${ϒ}_{p}^{X}=\frac{\partial X}{\partial p}\times \frac{p}{X}.$$

The sensitivity indices of $E^{⁎}$ (presented in (3)) and the $R_{0}$ were calculated and evaluated using parameter values in Table 1 and presented in Table 2. It is observed from Table 2 that, all parameters but two ($\beta_{1}$ and $\beta_{2}$) have an inverse relationship with the basic alcohol-abuse initiation number ($R_{0}$). Thus, increasing (or decreasing) these parameters leads to a decrease (or an increase) in $R_{0}$. Similarly, increasing (or decreasing) $\beta_{1}$ and $\beta_{2}$ leads to an increase (or a decrease) in $R_{0}$. The parameters with the most affect on $R_{0}$ are *θ* followed by $\beta_{1}$ and then $\beta_{2}$. Thus, in order to reduce $R_{0}$, efforts should be made to increase *θ* and reduce $\beta_{1}$ and $\beta_{2}$. As expected, increased $R_{0}$ is associated with disease progression and hence the relationship between $R_{0}$ and model parameters are expected to be the same for the infected compartments and model parameters. This is illustrated by the positive signs of the sensitivity indices of $R_{0}$, $M^{⁎}$ and $H^{⁎}$ with respect to $\beta_{1}$ and $\beta_{2}$. A counter-intuitive result is however observed for *μ* which is observed to have an inverse relationship with $R_{0}$ but a positive relationship with $M^{⁎}$ and $H^{⁎}$. Also the sensitivity index with respect to $\delta_{1}$ seems to be counter-intuitive in the case of $M^{⁎}$. However, this can be explained as follows: An increase in alcohol-induced deaths ($\delta_{1}$) among heavy-drinkers reduces the population of heavy drinkers and consequently the basic initiation number. This is likely to lead to more and more people changing their behavior towards heavy drinking to moderate drinking, causing the number of moderate drinkers to increase.

**Table 2:** Sensitivity indexes of $R_{0}$ and state variables of at the endemic equilibrium.

Model	Sensitivity Indices					
Parameters	$R_{0}$	*P*^⁎^	*M*^⁎^	*H*^⁎^	*C*^⁎^	*R*^⁎^
*α*_1_	-0.146	0.183	-0.056	-0.924	0.076	-0.056
*α*_2_	-0.007	0.060	-0.037	-0.080	-0.080	0.052
*α*_3_	-0.001	0.001	-0.001	-0.001	-0.001	-0.001
*b*	0.000	1.000	1.000	1.000	1.000	1.000
*β*_1_	0.832	-1.041	0.319	0.319	0.319	0.319
*β*_2_	0.168	-0.211	0.065	0.065	0.065	0.065
*δ*_1_	-0.015	-0.034	0.029	-0.058	-0.058	-0.058
*δ*_2_	0.000	-0.344	0.225	0.225	-0.431	-0.345
*γ*_1_	0.000	0.335	-0.225	-0.225	-0.553	0.358
*γ*_2_	0.000	0.033	-0.049	-0.049	-0.049	-0.862
*μ*	-0.001	-1.022	0.049	0.047	0.032	-0.154
*θ*	-0.831	1.039	-1.317	-0.317	-0.317	-0.317

**Figure 3:**
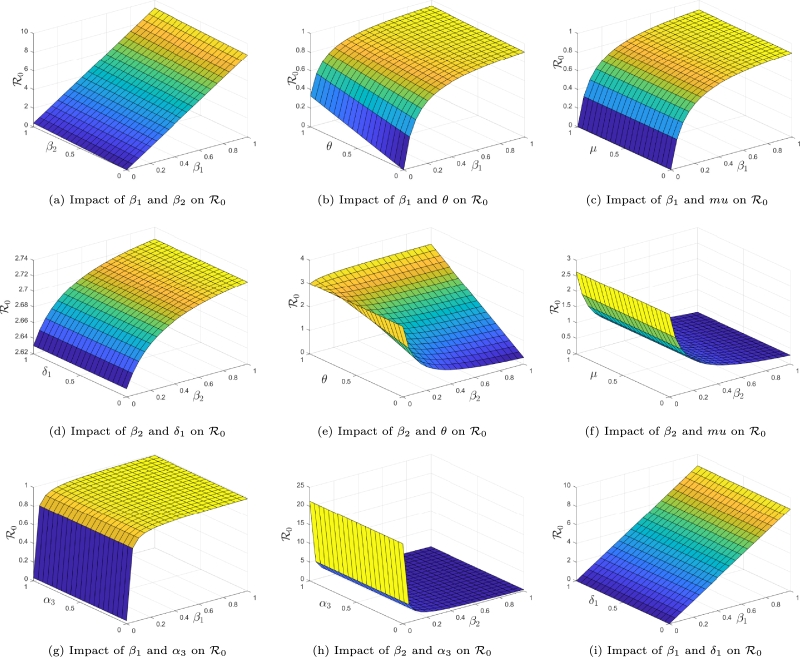
Surface plots showing the relationship between $R_{0}$ and some model parameters.

The relationship between some model parameters and $R_{0}$ using surface plots are depicted in Fig. 3.

Fig. 3 graphically shows how the model parameters (in pairs) influence the basic alcohol-consumption-initiation number. It is observed in Fig. 3a that $R_{0}$ linearly depends on the probabilities of adopting alcohol consumption behaviors due to contact with moderate drinkers ($\beta_{1}$) and heavy drinkers ($\beta_{2}$). As these parameters increase (decrease), the average number of new alcohol consumers will increase (respectively decrease). The other parameters are observed to have a nonlinear impact on $R_{0}$ (see Figs. 3b, 3c, 3d, 3e, 3f, 3g, 3h, and 3i). This suggests that, attempts to control the dynamics of alcoholism through vary these parameters needs a closer look than doing so by varying $\beta_{1}$ and $\beta_{2}$.

## 4 Development of the non-standard finite difference scheme (NFDS)

Numerical schemes used to solve differential equations are many and are often chosen based on some conditions which may include convergence, speed and ease of application. The most used method for solving ordinary differential equations models is the fourth-order Runge-Kutta method. This method is part of a family of explicit methods which have been shown to have inherent challenges such as poor stability and inefficiency. To circumvent these challenges, implicit numerical schemes may be used. In this section, a non-standard finite difference scheme that will be used to solve the system is developed (see [15] for further details on the method).

Since, all equations in (1) are in $C^{1}\left(R\right)$, the time derivatives are approximated using the forward difference scheme given by
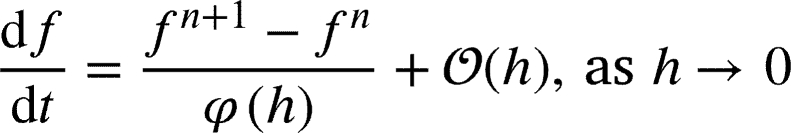
 In the equation above, the function φ(h) is called the denominator function, which must satisfy $\varphi \left(h\right)=1-e^{-h}$.

The model (1) can be approximated as follows:

(6) $$\begin{array}{c} \frac{P^{n+1}-P^{n}}{h}=b+\gamma_{2}R^{n}-\left(\frac{\beta_{1}M^{n}+\beta_{2}H^{n}}{N^{n}}+\mu \right)P^{n+1},\\ \frac{M^{n+1}-M^{n}}{h}=\left(\frac{\beta_{1}M^{n+1}+\beta_{2}H^{n}}{N^{n}}\right)P^{n+1}-\xi_{1}M^{n+1},\\ \frac{H^{n+1}-H^{n}}{h}=\theta M^{n+1}-\xi_{2}H^{n+1},\\ \frac{C^{n+1}-C^{n}}{h}=\alpha_{1}H^{n+1}-\xi_{3}C^{n+1},\\ \frac{R^{n+1}-R^{n}}{h}=\alpha_{2}H^{n+1}+\alpha_{3}M^{n+1}+\gamma_{1}C^{n+1}-\xi_{4}R^{n+1}. \end{array}\}$$

Rearranging the equations in (6) gives;

(7) $$\begin{array}{c} P^{n+1}=\frac{P^{n}+h\left(b+\gamma_{2}R^{n}\right)}{\left(1+h\mu +h\frac{\beta_{1}M^{n}+\beta_{2}H^{n}}{N^{n}}\right)},\\ M^{n+1}=\frac{\left(M^{n}+\frac{h\beta_{2}H^{n}P^{n+1}}{N^{n}}\right)}{\left(1+h\xi_{1}-h\frac{\beta_{1}P^{n+1}}{N^{n}}\right)},\\ H^{n+1}=\frac{1}{1+\xi_{2}h}\left(H^{n}+h\theta M^{n+1}\right),\\ C^{n+1}=\frac{1}{1+\xi_{3}h}\left(C^{n}+h\alpha_{1}H^{n+1}\right),\\ R^{n+1}=\frac{1}{1+h\xi_{4}}\left(R^{n}+h\left(\alpha_{2}H^{n+1}+\alpha_{3}M^{n+1}+\gamma_{1}C^{n+1}\right)\right). \end{array}\}$$

Now, since the equations in (7) have factors of the form 1+αh, we choose denominator functions of the form $\varphi (h,\alpha )=\left(1-e^{-\alpha \;h}\right)/\alpha$ so that the NFDS above can be written as follows [14]:

(8) Pn+1=Pn+φ1(h)(b+γ2Rn)(1+φ1(h)(μ+β1Mn+β2HnNn)),φ1(h)=1−e−hμμMn+1=(Mn+β2φ2(h)HnPn+1Nn)(1+φ2(h)ξ1−φ2(h)β1Pn+1Nn),φ2(h)=1−e−hξ1ξ1Hn+1=Hn+θφ1(3)Mn+11+ξ2φ3(h),φ3(h)=1−e−hξ2ξ2Cn+1=(Cn+α1φ4(h)Hn+1)1+ξ3φ4(h),φ4(h)=1−e−hξ3ξ3Rn+1=(Rn+φ5(h)(α2Hn+1+α3Mn+1+γ1Cn+1))1+φ5(h)ξ4,φ5(h)=1−e−hξ4ξ4n=0,1,2…P1=P(0),M1=M(0),R1=R(0),H1=H(0),C1=C(0),R1=R(0).}

## 5 Numerical results and discussions

In this section, the non-standard finite difference scheme (8) developed in section 4 is used to solve the model (1). The essence of the numerical solution is to illustrate the analytical findings and also to conduct a deeper examination of how model parameters influence the prevalence of alcohol abuse within the population. The model parameter values in Table 1 were used to carry out the simulations.

**Figure 4:**
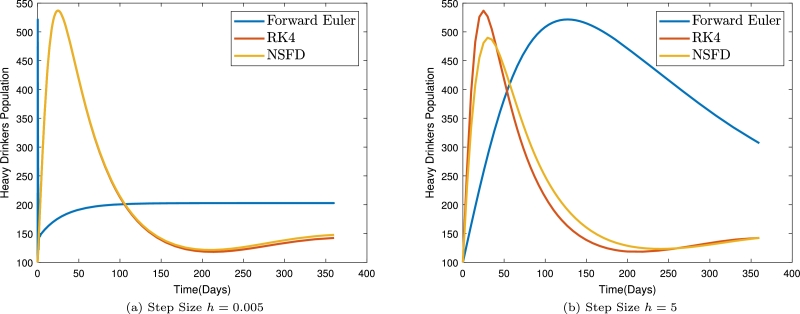
Plots of heavy drinkers population using the NSFDS and two other numerical schemes for different steps sizes.

To illustrate the superiority of the non-standard finite difference scheme, we solved the model using three different methods; The forward Euler method, the Runge-Kutta fourth order scheme, and the developed NSFDS. The results are presented in Fig. 4. It is observed that the developed non-standard finite difference scheme outperforms the Euler's scheme both for large step sizes (see Fig. 4b) and very small step sizes (see Fig. 4a). The results for our method and the RK method are not so different. However, we note that our method is computationally cheaper than the RK method. This gives us a good reason to adopt our developed method for the simulation.

**Figure 5:**
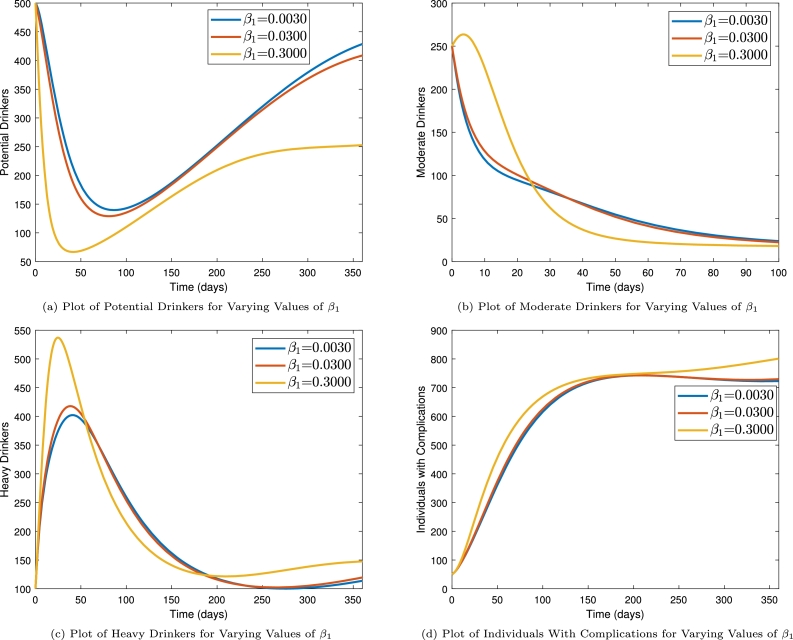
Time series plots showing the impact of alcohol-abuse transference (*β*_1_) on population dynamics.

Fig. 5 illustrates the impact of alcohol abuse behavior adoption caused by the interaction between potential and moderate drinkers. To study this, we varied the parameter $\beta_{1}$ and plotted the results for the various values of the transmission parameters. From Fig. 5a, it is observed that increasing $\beta_{1}$ leads to decrease in the potential drinkers population. This is as a result of the fact that increasing $\beta_{1}$ increases the chance of people adopting alcohol consumption behaviors and subsequently the population of the potential drinkers. It is also observed that, increasing the probability of acquiring drinking habits through interaction with drinkers leads to increase in the number of moderate drinker (see Fig. 5b) and heavy drinkers (see Fig. 5c) and subsequently the number of individuals who develop alcohol-consumption-related complications (see Fig. 5d). The dynamics are however observed to switch for a while after some time where the number of drinkers is reduced for increased values of $\beta_{1}$. This switch can be attributed to the fact that as more individuals develop complications the number of drinkers available to introduce potential drinkers into alcohol consumption will reduce and consequently the number of drinkers will reduce. This explains why it may be impossible for alcoholism to be adopted by the whole population.

**Figure 6:**
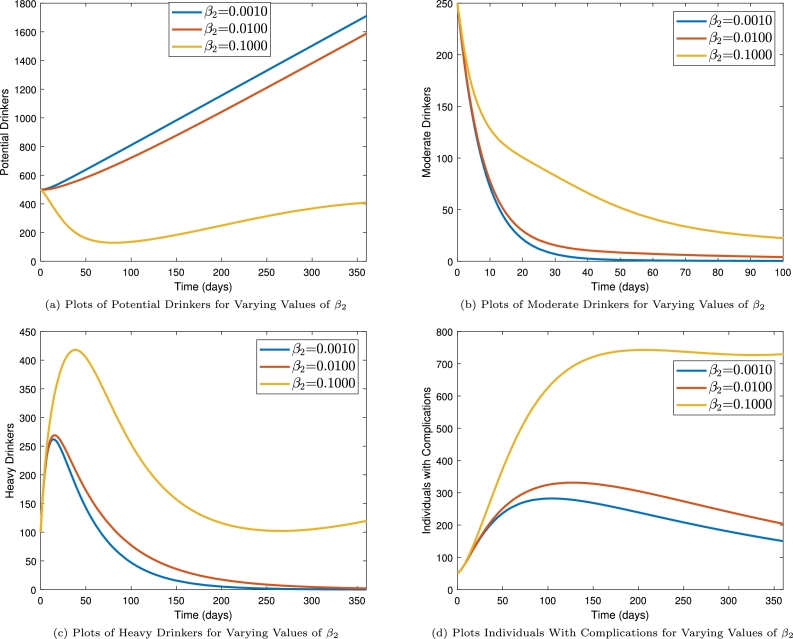
Time series plots showing the impact of alcohol abuse infections on alcohol consumer populations.

The impact of probability of alcohol-abuse adoption caused by heavy drinkers is presented in Fig. 6. We observe that increasing the probability of alcohol-consumption transference from heavy drinkers increases the moderate drinkers (see Fig. 6b), heavy drinkers (see Fig. 5c) and individuals with complications (see Fig. 6d). These increases in the alcohol consumers populations lead to a corresponding decrease in the potential drinkers population (see Fig. 6a) as $\beta_{2}$ increases. We therefore note that, efforts should be made to keep the chances of individuals involved in alcoholism influencing others from engaging in the habit of drinking. This is more especially important in the case of minors in certain parts of the developing world who may often be sent on errands to buy alcoholic beverages. It is found in Table 2 that when the rates of recoveries ($\alpha_{2}$ and $\alpha_{3}$) are increased, the basic initiation number decreased. This implies that, moderate and heavy drinkers should be given the necessary support in order to get them fully recovered. This will go a long way to curb the spread of alcohol abuse infections in the society.

**Figure 7:**
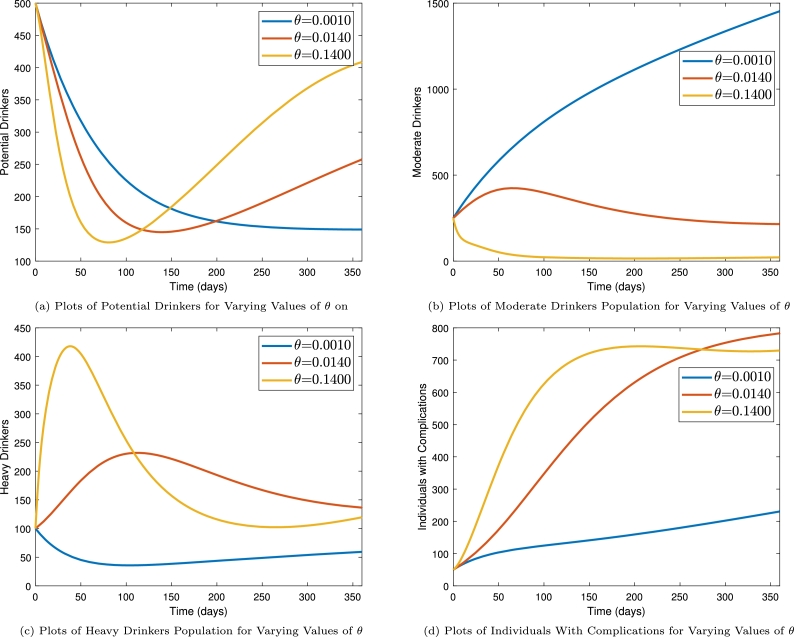
Time series plots showing the impact of transiting from moderate drinking to heavy drinking on population dynamics.

The impact of the rate of transition from moderate to heavy drinkers was also studied by solving the model for varying values of the associated parameter, *θ*. It can be observed from Fig. 7 that, when (*θ*) is increased, the number of potential drinker and moderate drinkers decrease (see Fig. 7a and Fig. 7b) while the number of heavy drinkers and those with complications increase (see Fig. 7c and Fig. 7d). Thus, measures need to be taken to ensure that moderate drinking does not progress into heavy drinking. This can be achieved by ensuring that the frequency of drinking be reduced to the barest minimum. Also, the phenomenon of resorting to drinking to overcome problems or stress need to be curbed through counseling as that has the potential of resulting into heavy drinking if the problems persist.

## 6 Conclusions

In this paper, we put forth a nonlinear differential equations model to elucidate the dynamics of alcoholism in a population. The population is split into five mutually-exclusive compartments of Potential drinker's moderate drinkers, heavy drinkers and individuals who have developed complications due to excessive consumption of alcohol. The model is developed by treating alcoholism as a contagion and following the theory of disease transmission from infected persons to Susceptible persons, consider the process of drinkers influencing non-drinkers as a transmission using standard incidence function. We adopted a recently developed technique [17] to determine basic alcohol-abuse initiation number which is similar to the basic reproduction number to describe the average number of secondary alcohol consumers that are initiated into alcoholism by a single alcoholic that is introduced into an otherwise completely non-drinkers population. The alcohol-abuse-free equilibrium is shown to be globally asymptotically stable whenever the basic alcohol-abuse initiation number is not more that 1. This global stability results was further confirmed by the existence of forward bifurcation in the model at $R_{0}=1$. The sensitivity analysis revealed that, the most sensitive parameter to the $R_{0}$ is rate of transition into heavy drinking *θ* followed by probability of initiation per contact with a moderate drinker $\beta_{1}$ and then the probability of initiation per contact with a heavy drinker $\beta_{2}$. In order to conduct simulation on the model, we developed and adopted an implicit non-standard numerical scheme which overcomes the inherent challenges of standard explicit schemes. The numerical simulations confirmed that, the spread of alcohol infections and alcohol-induced complications can be minimized by lowering the probabilities of initiation per contact between potential drinkers and alcohol abusers.

There are multiple potential extensions of this study: The incidence functions have been shown to have interesting effect on the dynamics of models. While this work adopted a standard incidence function to describe initiation into alcoholism, future research could adopt saturated incidence functions as in [20], [21]. Recently, fractional and fractional-fractal operators have been adopted in the development epidemic models. These models have been said to be more realistic than their integer-order counterparts due to the inherent memory property the fractional differential operator. The current can be extended by incorporating fractional and fractal-fractional operators as in [22], [23]. Also, a discrete-time model versions of the model can be studied as in [24]. It is hoped that considering these extensions in future studies will improve our understanding of the dynamics of alcohol consumption and its related effects.

## CRediT authorship contribution statement

**Eric Abaa Baba Sandow:** Writing – original draft, Methodology, Formal analysis, Data curation, Conceptualization. **Baba Seidu:** Writing – original draft, Formal analysis, Data curation, Conceptualization. **Stephen Abagna:** Writing – review & editing, Formal analysis.

## Declaration of Competing Interest

The authors declare that they have no known competing financial interests or personal relationships that could have appeared to influence the work reported in this paper.

## Data Availability

No data was used for the research described in the article.
